# Subbasal corneal nerve damage in patients with bacterial keratitis: in vivo confocal microscopy study

**DOI:** 10.5935/0004-2749.20220010

**Published:** 2022

**Authors:** Rodrigo Thiesen Müller, Beatriz B. de Andrade, Luciene Barbosa de Sousa

**Affiliations:** 1 Department of Ophthalmology and Visual Sciences, Escola Paulista de Medicina, Universidade Federal de São Paulo, São Paulo, SP, Brazil; 2 Hospital de Olhos de Blumenau, Blumenau, SC, Brazil

**Keywords:** Cornea/innervation, Ophthalmic nerve, Eye infections, viral, Keratitis, herpetic, Microscopy, confocal, Córnea/inervação, Nervo oftálmico, Infecções ocu lares virais, Ceratite herpética, Microscopia confocal

## Abstract

**Purpose:**

To examine subbasal corneal nerve changes in patients with bacterial infectious keratitis using in vivo confocal microscopy.

**Methods:**

Thirteen patients (13 eyes) with unilateral bacterial keratitis and 12 healthy controls were prospectively enrolled in the study. In vivo confocal microscopy was performed in all the patients at 2 time points, in the acute phase of infectious keratitis and at 28 ± 0.6 months after resolution of the infection.

**Results:**

The subbasal nerve length was 5.15 ± 1.03 mm/mm^2^ during the acute phase of bacterial keratitis (compared with that of the controls: 19.02 ± 1.78 mm/mm^2^, p<0.05). Despite the significant corneal nerve regeneration over the interval of 28 months after the resolution of the infection, the nerve density was still significantly reduced as compared with that of the controls (9.73 ± 0.93 mm/mm^2^, p<0.05). Moreover, in vivo confocal microscopy images showed diffuse high-reflecting areas referring to the scar tissue areas with thin and tortuous nerve branches regenerating toward these areas.

**Conclusions:**

A partial corneal nerve regeneration of subbasal nerve plexus during the first 28 months after the acute phase of infectious keratitis was observed. Moreover, the regenerated nerves of the patients remained morphologically altered as compared with those of the healthy controls. These results may be relevant to the clinical follow-up and surgical planning for these patients.

## Abstract (pt)

**Objetivo:**

Relatar as alterações no plexo nervoso corneano subbasal em pacientes com ceratite infecciosa de origem bacteriana utilizando a microscopia confocal in vivo.

**Métodos:**

Treze olhos de 13 pacientes com ceratite bacteriana unilateral e 12 indivíduos saudáveis como grupo controle foram incluídos prospectivamente no estudo. A microscopia confocal in vivo foi realizada em todos os pacientes em 2 momentos: na fase aguda da ceratite infecciosa e após 28 ± 0,6 meses da resolução da infecção.

**Resultados:**

A densidade dos nervos no plexo subbasal foi de 5,15 ± 1,03 mm/mm^2^ na fase aguda da ceratite infecciosa (comparada com o grupo controle: 19,02 ± 1,78 mm/mm^2^, p<0,05). Apesar de significativa regeneração dos nervos corneanos ao longo de um intervalo de 28 meses após a resolução da infecção, a densidade dos nervos se manteve significativamente reduzida (9,73 ± 0,93 mm/mm^2^) quando comparada com o grupo controle (19,02 ± 1,78 mm/mm^2^, p<0,05). Além disso, as imagens obtidas com a microscopia confocal mostraram áreas de hiperreflectividade referente ao tecido corneano cicatricial com ramos de nervos, afinados e tortuosos, se regenerando nessas áreas.

**Conclusões:**

Foi observado regeneração parcial dos nervos do plexo corneano subbasal durante os primeiro 28 meses após a resolução da fase aguda da ceratite infecciosa. Além disso, os nervos corneanos regenerados se mantiveram morfologicamente alterados quando comparados ao grupo controle. Esses resultados podem ser relevantes para o acompanhamento clínico e planejamento cirúrgico desses pacientes.

## INTRODUCTION

Infectious keratitis (IK) is an important cause of visual impairment and blindness worldwide^(1,2)^. The disorder can progress rapidly and requires immediate treatment to minimize vision threatening and complications^(1)^. Cariello et al. previously reported that nearly 40% of cultures of corneal scrapings of patients with IK were positive for bacteria^(3)^. Bacterial keratitis is painful and often induces severe corneal inflammation and opacities. Corneal opacities are the fourth leading cause of blindness globally and responsible for 10% of avoidable visual impairments in the world’s least developed countries^(2)^.

The human cornea is one of the most densely innervated tissues of the body. Cornea innervation is supplied by the long ciliary nerve and terminal branches of the ophthalmic division of the trigeminal nerve^(4-6)^. Corneal innervation has important trophic functions and plays an important role in the regulation of epithelial integrity, proliferation, and wound healing^(7,8)^. The corneal ulceration and subsequent scarring caused by IK may result in corneal nerve damage with clinical consequences due to impaired corneal nerve function^(9)^.

Laser in vivo confocal microscopy (IVCM) is a noninvasive and high-resolution imaging modality^(10)^. This technology can aid in the in vivo assessment of diseases at the cellular level, providing images comparable with histochemical methods. In recent years, the use of IVCM has revealed the importance of corneal nerves in both healthy eyes and ocular diseases, including IK, cornea transplantations, laser keratorefractive surgeries, neurotrophic keratopathy, and dry eye disease^(5,11,12)^.

A decrease in corneal nerves and an increase in immune dendritic cells in patients with microbial keratitis have been demonstrated in previous studies^(5,9,13)^. However, the present study evaluated corneal nerve changes in patients with IK over a long period beyond the acute phase (>24 months) by using IVCM. This study postulates that changes in the subbasal nerve layer may be severe enough to persist even after a long period from the acute infectious phase.

## METHODS

This was a prospective, longitudinal, single-center study that used a controlled and single-blind method. Thirteen patients who had unilateral IK treated at the Cornea Service of the Blumenau Eye Hospital (Blumenau, SC, Brazil) between 2016 and 2017 were included in the study. Twelve ageand sex-matched volunteers were included as healthy controls. The diagnosis of acute IK was made by a cornea specialist on the basis of clinical history and ophthalmological examination. Only patients with bacterial etiology were included in the study on the basis of positive corneal cultures or good treatment response to antibiotics eye drops. The study excluded subjects with IVCM findings consistent with fungal or Acanthamoeba keratitis, in addition to a history of prior IK, ocular inflammatory disease, ocular trauma, previous eye surgery, and diabetes.

### Laser IVCM

Laser IVCM (Heidelberg Retina Tomograph 3 with the Rostock Cornea Module [HRT3/RCM]; Heidelberg Engineering GmbH, Heidelberg, Germany) was performed in all the patients and controls. A 670-nm red wavelength diode laser source equipped with a ×63 objective immersion lens with a numerical aperture of 0.9 (Olympus, Tokyo, Japan) was used. The laser confocal microscope provides images that represent a coronal section of the cornea of 400 × 400 µm, which is 160,000 µm^2^, providing ×800 magnification of the corneal tissue.

A disposable sterile polymethylmethacrylate cap (Tomo-Cap; Heidelberg Engineering GmbH) filled with a layer of lubricating gel (2 mg/g carbomer Vidisic gel; Bausch & Lomb, Heidelberg, Germany) was mounted in front of the cornea module optics for each examination. One drop of topical anesthesia 0.5% proximetacaine (Anestalcon; Alcon, SP, Brazil) was instilled in both eyes, followed by a drop of lubricating gel.

### Image analysis

**Figure 1:**
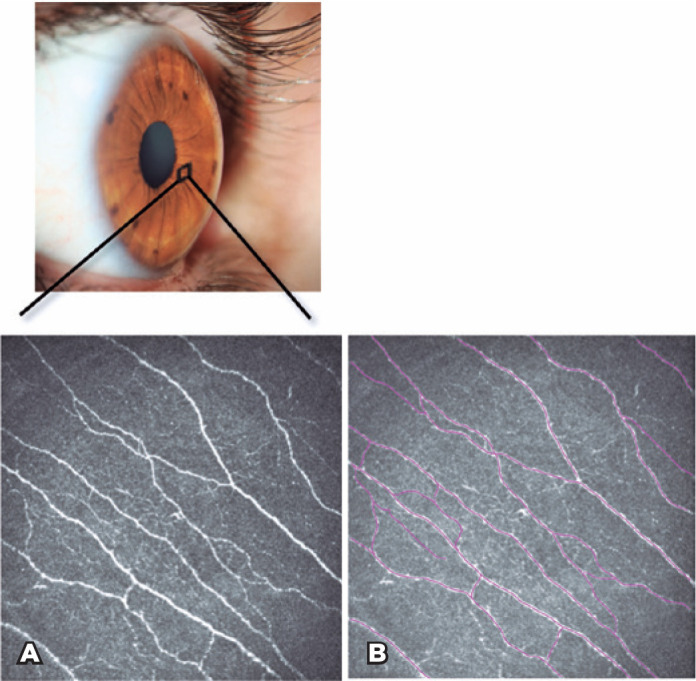
A) In vivo confocal microscopy image of a healthy control showing subbasal corneal nerve plexus. B) Subbasal nerve fiber density measurements traced (purple tracings) using the Neuron J software.

Subbasal nerve fiber density measurements were traced using the NeuronJ software (http://www.imagescience.org/meijering/software/neuronj/), which is a semi-automated nerve analysis plug-in of ImageJ that traces all visible nerve fibers in the image and calculates their total length in millimeters (Figure 1). The subbasal nerve densities (nerve length and number of nerves) were categorized as main nerve trunks, nerve branches, and total nerves. Three best-focused images with good contrast from each area were selected for subbasal corneal nerve analysis. The entire image had to be localized at the subbasal layer, which was immediately at or posterior to the basal epithelial layer and anterior to the Bowman layer. The IVCM images were obtained at 2 time points, in the acute phase of IK (corneal ulcer phase) and at the interval time of 28.3 ± 0.6 months after the resolution of the infection (corneal scar phase). Two independent masked observers analyzed all the images.

### Statistical analysis

Statistical analysis was performed using Stata version 13.0 (Stata Corp, TX, USA). Continuous variables were expressed as mean ± standard error of the mean, whereas categorical variables were described by frequency and percentage, unless otherwise indicated. The normality of the data was investigated using the Shapiro-Wilk test. A chi-square test was used to compare the qualitative variables. The Wilcoxon signed-rank test (for paired groups) or Kruskal-Wallis (for unpaired groups) were used to assess differences between visits or groups. The Bonferroni post hoc test was used for further analysis, when appropriate. Correlations were analyzed using the Spearman rank correlation test. For each test, a p value of <0.05 was considered significant.

## RESULTS

Thirteen patients (5 men and 8 women) with unilateral bacterial keratitis and 12 controls (5 men and 7 women) were enrolled in the study. The mean ages of the patients and controls were 35.8 ± 3.6 and 40.9 ± 2.6 years, respectively. No statistically significant differences in the characteristics were found between the groups (p>0.05 for all). The corneal ulcer diameter was <2 mm in 4 patients and >2 mm in 9 patients. The duration of treatment using antibiotic drops was 21.6 ± 2.0 days. The interval time between the study visits for the IVCM image acquisitions was 28.3 ± 0.6 months. The demographic and clinical characteristics are summarized in table 1.

**Table 1:** Demographic and clinical characteristics of the patients with infectious keratitis and the healthy controls

	Control group	Infectious keratitis group
Number of patients (n)	12	13
Sex, male/female (n)^*^	5/7	5/8
Age (years)^*^	40.9 ± 2.6	35.8 ± 3.6
Corneal ulcer diameter, <2 mm/>2 mm (n)	-	4/9
Treatment duration (days)	-	21.6 ± 2.0

Values are presented as mean ± SEM, unless otherwise indicated.

* No statistically significant difference between the groups.

### Subbsal corneal nerve analysis using IVCM

#### Nerve alterations during the acute phase of bacterial keratitis

**Figure 2:**
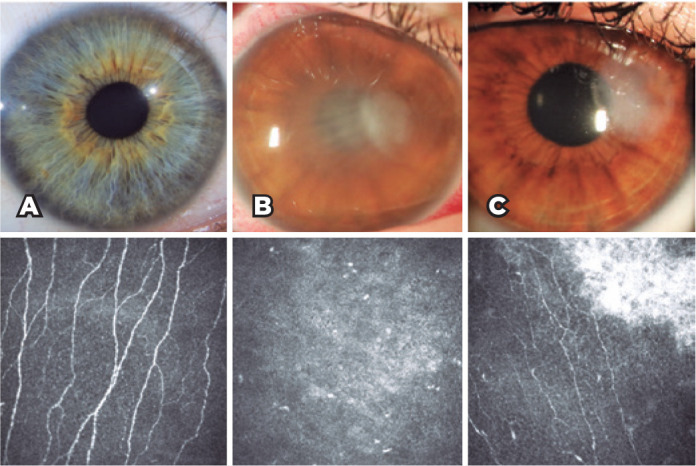
Slit-lamp photographs and in vivo confocal microscopy (IVCM) images for evaluation of the corneal subbasal nerves in the healthy control and patient groups. A) Normal subbasal nerve fibers in the healthy control group. B) IVCM image of an eye with infectious keratitis during the acute phase. C) The same eye after 2 years of follow-up shows corneal nerve fibers regenerating near the residual corneal scar tissue.

**Figure 3:**
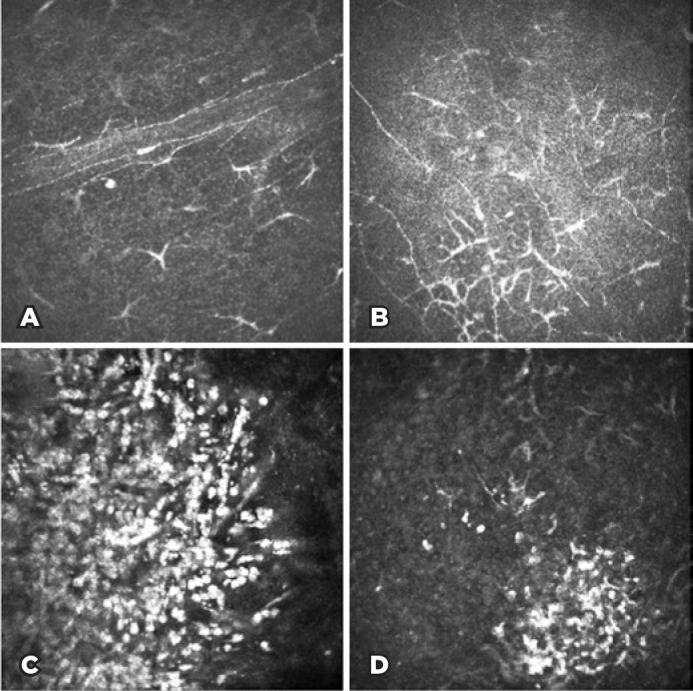
In vivo confocal microscopy (IVCM) images during the acute phase of infectious keratitis. A and B) Decrease in subbasal nerve density and increase in the number of dendritiform cells. C and D) Immune cell infiltration related to the acute phase of inflammation of the infectious disease.

The total subbasal nerve length was 5.15 ± 1.03 mm/mm^2^, and the total number of subbasal nerves was 3.31 ± 0.62 nerves/frame during the acute phase of bacterial keratitis (p<0.05 for all, compared with the controls: 19.02 ± 1.78 mm/mm^2^ and 21.80 ± 1.31 nerves/frame; Figure 2). In addition to the significant reduction in the nerve density of the subbasal plexus, massive immune cell infiltration at the basal epithelial and subbasal levels was observed in the IVCM images. These cells are characterized by high-reflecting dendritiform structures composed of body and dendrites (dendritic cells) in addition to their bright round shape structures (polymorphonuclear cells). A representative IVCM image taken during the acute phase can be seen in figure 3.

#### Nerve alterations in corneal scar due to bacterial keratitis

**Figure 4:**
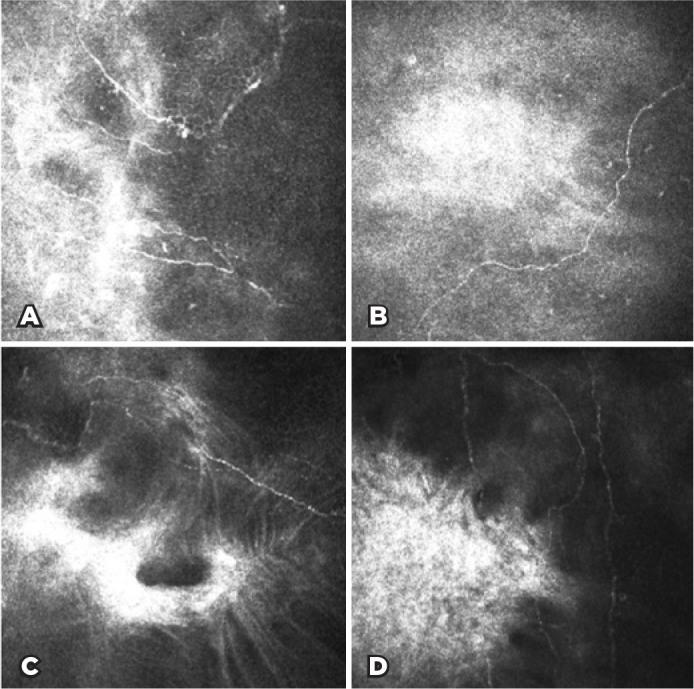
In vivo confocal microscopy (IVCM) images after an average of follow-up period of 28 months in the patients with infectious keratitis. A-D) Subbasal nerves regenerate toward the corneal scar tissue but more thinly and tortuously and less densely than in the healthy control group.

The total subbasal nerve length was 9.73 ± 0.93 mm/mm^2^, and the total number of subbasal nerves was 9.77 ± 0.80 nerves/frame during the corneal scar phase (p<0.05 for all, compared with the controls: 19.02 ± 1.78 mm/mm^2^ and 21.80 ± 1.31 nerves/frame; Table 2). Moreover, the IVCM images showed diffuse high-reflecting areas referring to the scar tissue areas with thin and tortuous nerve branches regenerating toward these areas (Figure 4).

**Table 2:** Corneal subbasal nerve parameters in the control and bacterial keratitis groups

	Control group	Bacterial keratitis group	
		Corneal ulcer phase phase	Corneal scar
Total nerve length, mm/mm^2^	19.02 ± 1.78	5.15 ± 1.03^*^	9.73 ± 0.93^* †^
Main nerve trunk length, mm/mm^2^	9.66 ± 0.93	1.48 ± 0.37^*^	2.98 ± 0.27^* †^
Nerve branch length, mm/mm^2^	9.36 ± 0.91	3.66 ± 1.01^*^	6.75 ± 0.79^* †^
Total nerve number, n/frame	21.80 ± 1.31	3.31 ± 0.62^*^	9.77 ± 0.80^* †^
Number of main nerve trunks, n/frame	3.92 ± 0.29	0.85 ± 0.09^*^	1.81 ± 0.17^* †^
Nerve branches numbers, n/frame	17.88 ± 1.21	2.46 ± 0.60^*^	7.96 ± 0.69^* †^

Data are expressed as mean ± standard error of the mean.

* Statistical significance in comparison with the controls.

† Statistical significance in comparison with the corneal ulcer phase.

**Figure 5:**
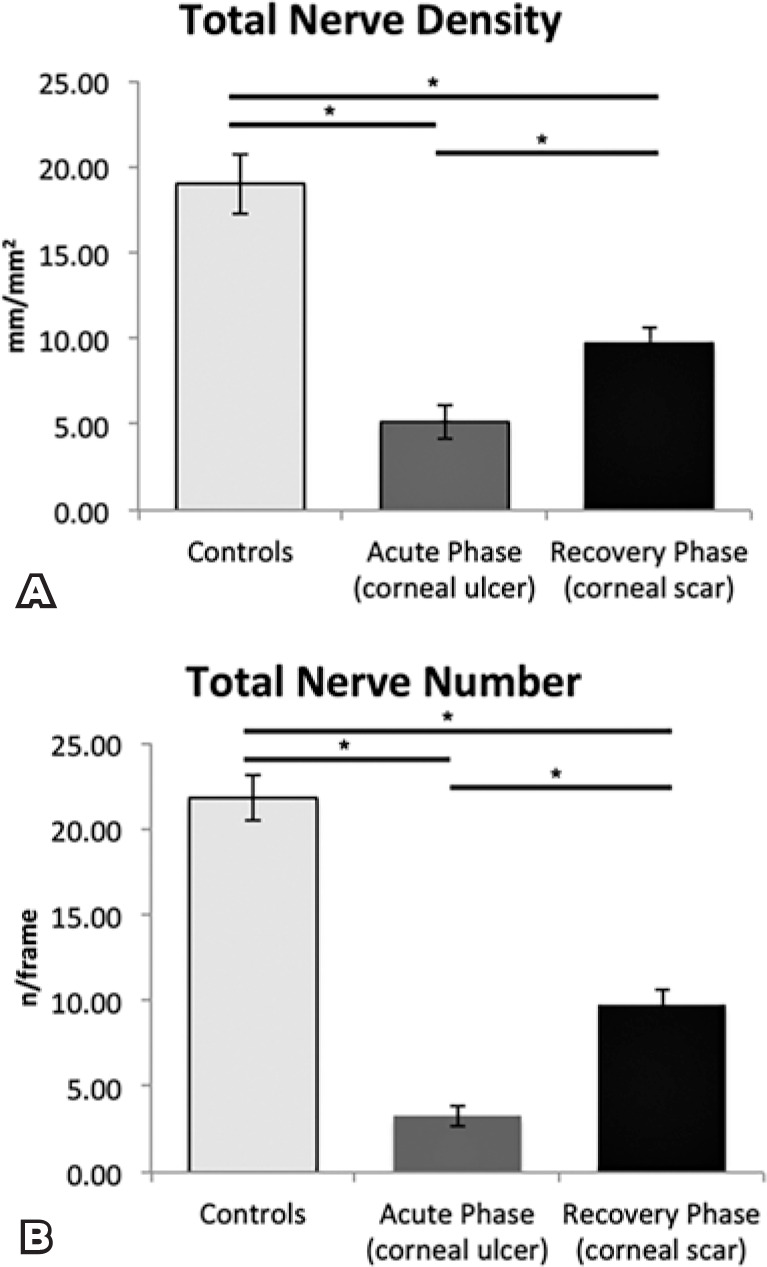
The bar graphs show subbasal corneal nerve alterations in the healthy control and infectious keratitis groups. A) Corneal nerves regenerated over the interval time, but the total nerve density was still significantly reduced as compared with that in the controls. B) The total number of nerves was significantly reduced during the acute phase and did not reach normal levels after the follow-up period. The bars indicate the mean ± standard error of the mean. *p<0.05.

The total subbasal nerve length and total number of nerves during the corneal scar phase showed significant increases as compared with their values in the acute phase (5.15 ± 1.03 mm/mm^2^ vs. 9.73 ± 0.93 mm/mm^2^ and 3.31 ± 0.62 nerves/frame vs. 9.77 ± 0.80 nerves/ frame, respectively; p<0.05 for all; Figure 5).

Despite the significant corneal nerve regeneration over the interval time, the total nerve length and total number of nerves were still significantly reduced as compared with the controls (p<0.05 for all; Table 2). The correlation analysis did not show statistically significant correlations between nerve density and the following: age, sex, corneal ulcer diameter, and treatment duration.

## DISCUSSION

This study revealed persistence of nerve damage at the level of the cornea subbasal layer, even after 2 years of resolution of the bacterial cornea infection. Hamrah et al. published a significant decrease in subbasal nerve fiber density in keratitis caused by both the herpes simplex virus (HSV) and varicella zoster virus (VZV), strongly correlating with the reduction in corneal sensation^(14)^. In addition, other prospective studies demonstrated that the decrease in subbasal corneal nerve density is associated with increased density and morphological changes of central epithelial dendritic cells in patients with IK, including bacterial, fungal, and Acanthamoeba keratitis, suggesting a potential direct interaction between the immune and nervous systems in the cornea^(5,15)^.

The human cornea receives a large supply of sensory nerves from the long ciliary nerve branch of the trigeminal nerve^(15)^. The corneal nerves and histological cellular and structural details of the cornea can be visualized with IVCM. The IVCM allows for systematic studies of subbasal corneal nerve parameters. This implement offers a quick result, noninvasiveness of the imaging procedure, and images of the corneal layers at the cellular level in real time^(12,16)^. The examination facilitates the investigation of many cornea surface diseases such as dry eye, IK, and subsequent evaluation of corneal transplantation^(17)^.

This prospective longitudinal study evaluates the corneal nerve changes in patients with IK over a mean follow-up time of >2 years. Subbasal corneal nerve impairment was found during the acute phase of the IK. Although we observed a statistically significant regeneration of nerves in these patients as compared with the acute phase, it remains diminished and morphologically altered as compared with the healthy control group. This result demonstrates a partial recovery of nerve fibers.

A profound decrease in corneal innervation in patients with IK (including bacterial, fungal, and Acanthamoeba keratitis) was already demonstrated in a previous study with subsequent partial corneal reinnervation in the first 6 months after resolution of the infection^(9)^. Besides that, corneal degeneration and nerve regeneration correlated with corneal esthesiometry.

Moreover, over a mean follow-up period of >3 years, Moein et al. observed a statistically significant regeneration of central corneal subbasal nerves in eyes affected by HSV keratitis^(18)^. Research groups have published an inverse correlation between dendritic cell and subbasal corneal nerve density in patients with IK, proposing an association between corneal immune response and the nervous system^(15)^. Dendritic cells are antigen-presenting cells positioned at the level of the basal epithelium and Bowman’s layer as immune sentinels.

The aim of the present study was to present quantitative and qualitative analyses of changes of the subbasal corneal nervous plexus at the cellular level by using images obtained with IVCM technology. However, this study has some limitations. Clinical data such as corneal esthesiometry values were not obtained over the course of the study. All the patients were treated with the best treatment available by using the most appropriate medications as defined by the corneal specialist. However, the use of different eye drops among the patients may potentially interfere with the nerve regeneration process. Future analyses should confirm the occurrence of clinical consequences in these patients with morphological corneal nerve alterations.

In conclusion, a partial corneal nerve regeneration of the subbasal nerve plexus was observed during the first 28 months after the acute phase of IK. Moreover, the regenerated nerves remained morphologically altered as compared with those in the healthy control group. These results may be relevant to clinical follow-up of and surgical planning for these patients. The decrease in the subbasal plexus may be a risk factor for the development of new corneal infection and dry eyes. Future studies should be performed to link and correlate IVCM findings and clinical outcomes in patients with IK.
